# Significance of stereologically spatiotemporal cells in molecular medicine

**DOI:** 10.1002/ctm2.70470

**Published:** 2025-09-16

**Authors:** Xuanqi Liu, Wanxin Duan, Yuyang Qiu, Ruyi Li, Yuanlin Song, Xiangdong Wang

**Affiliations:** ^1^ Department of Pulmonary and Critical Care Medicine, Zhongshan Hospital Fudan University Shanghai Medical College Shanghai China; ^2^ Shanghai Institute of Clinical Bioinformatics Shanghai China; ^3^ Fudan University Centre of Clinical Bioinformatics Shanghai China

**Keywords:** organelles, single‐cell, spatialization, stereology, temporalization

## Abstract

Spatiotemporal distributions of intracellular elements (e.g., small molecules, proteins and organelles) dynamically altered in response to extracellular stimuli and pathogens, regulating those element movements, remodelling, and functions independently of mere changes in element abundance. To distinguish from conventional one‐ or two‐dimensional spatialization, we define the precise three‐dimensional localisation and interactions of intra‐ and extracellular elements at the single cell level as the “stereologically spatiotemporal cell” (SST‐cell). For example, the three‐dimensional construction of chromosomes ensures their proper formation and spatial positioning, facilitates the recruitment of regulatory factors, and underlies the mechanisms by which these factors maintain chromatin architecture. A large number of intracellular organelles and sub‐organelles, along with their intercommunications, decide cellular biological types, subtype specification and type‐specific functions. With the development of Stereo‐Cell and Stereo‐seq, the measurement of spatial SST‐cell omics probably enables the detailed dissection of spatial heterogeneity among different cell subtypes and states, as well as their intercellular communications. Furthermore, the new approach of single SST‐cell drug screening will be innovated for developing the new generation of clinical precision therapies.

With the continuous development of biotechnology, our understanding of cells, the fundamental units of the human body, and their functions has deepened significantly. The structural and functional characteristics of cells are increasingly recognised as multidimensional and complex, shaped by their tissue and organ locations, intercellular connections, interactions in the extracellular fluids, and the dynamics of subcellular organelles and molecules. Cellular heterogeneity among cells primarily arises from variations in the extracellular microenvironment, intracellular genetic diversity, and the spatial and dynamic arrangement of subcellular components, including nuclei, organelles, molecules, and cytoplasm. Recent research has evidenced that a large proportion of proteins are spatially allocated in intracellular compartments, including membrane‐bound and membrane‐less organelles. This localisation forms protein‐driven spatial networks that link these organelles and orient interconnections among the compartments.^1^ These spatiotemporal distributions of proteins dynamically altered in response to extracellular stimuli and pathogens, regulating protein movements, remodelling, and functions independently of mere changes in protein abundance. Trans‐compartmental translocations of intracellular components orientate dynamic and multiple regulations of signalling and functions. Studies on the spatiotemporal dynamics of intracellular proteomic and phosphor‐proteomic signalling networks have demonstrated that receptor adaptor proteins can be redistributed among subcellular compartments. This can transition from a free cytosolic form to membrane‐bound fractions and be targeted to receptors through vesicles, a process activated by the phosphorylation of tyrosine residues in the receptor, like the interaction between epidermal growth factor and epidermal growth factor receptor.^2^ Various subcellular compartmentations are recognised and defined by specific biomarkers at a two‐dimensional level, which partly shows the spatialization and temporalization of compartments and molecular relocations in stereological cells (Figure 1).

**FIGURE 1:**
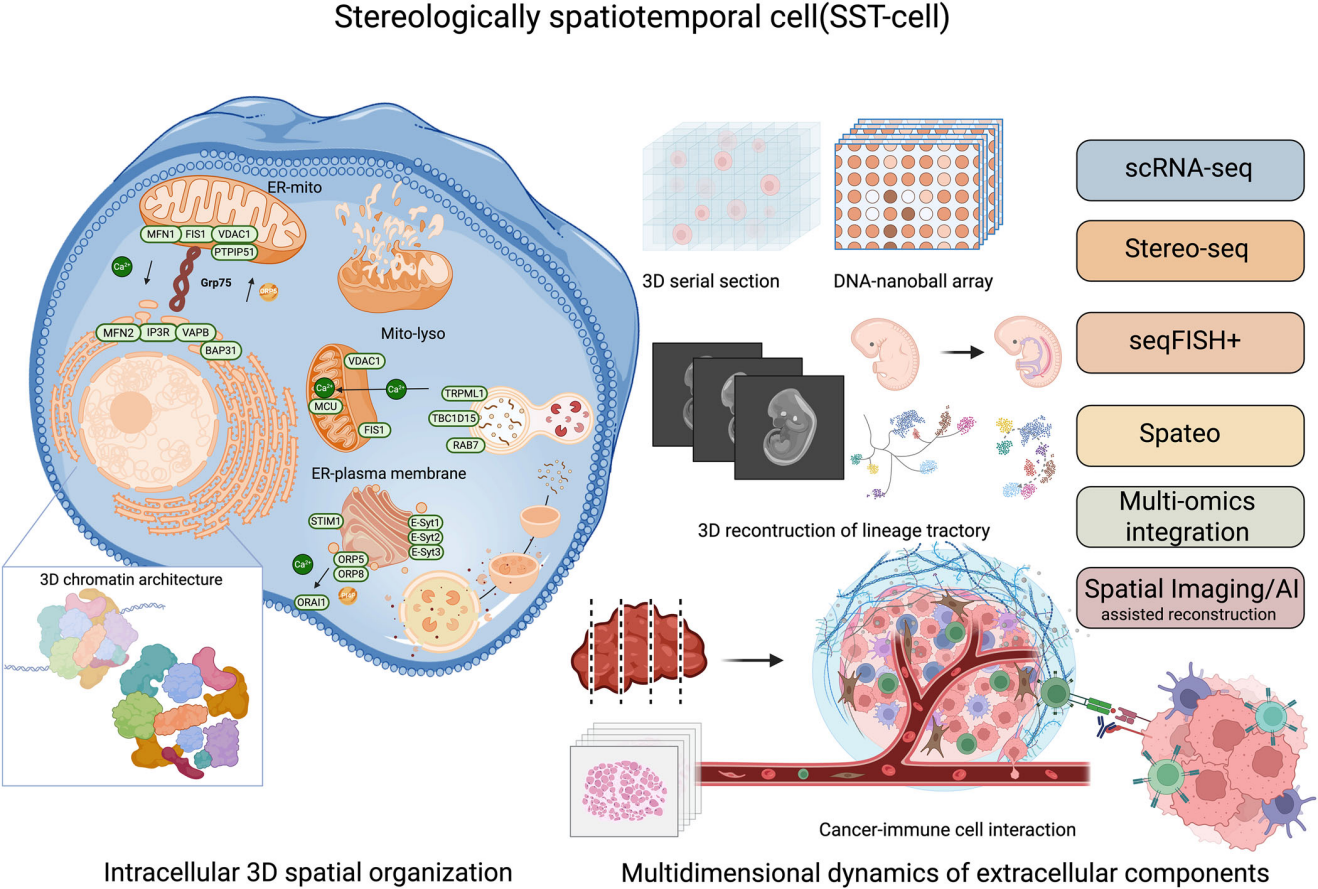
Conceptual framework of the stereologically spatiotemporal cell (SST‐cell): decoding intracellular and extracellular spatial dynamics at single‐cell resolution. This graphical abstract illustrates the emerging concept of the stereologically spatiotemporal cell (SST‐cell), defined as a functional unit with precise three‐dimensional (3D) spatial localisation and dynamic interactions of intra‐ and extracellular components. The SST‐cell integrates subcellular organisation, molecular dynamics, and extracellular microenvironmental cues to explain cellular heterogeneity and functional diversity in complex tissues. Intracellular 3D spatial organisation: Depicted is a single cell with key organelle–organelle contact sites, including the ER–mitochondria (ER–mito), mitochondria–lysosome (mito–lyso), and ER–plasma membrane (ER–PM) interfaces. The membrane‐contact sites (MCSs) mediate spatial signalling and material exchange, governed by molecular tethers such as MFN1/2, VDAC1, IP3R, TRPML1, RAB7, STIM1, ORP5/8 and associated regulatory proteins (e.g. Grp75, TBC1D15 and FIS1). Organelle interactomes dynamically respond to physiological stimuli and pathological insults by spatial rearrangement and trans‐compartmental protein trafficking. The inset highlights 3D chromatin architecture, emphasising the importance of spatial genome organisation in regulating gene expression, epigenetic control, and cellular identity. The chromatin conformation modulates accessibility of regulatory elements, nuclear compartmentalisation, and genome stability, with implications for development and disease. Multidimensional dynamics of extracellular components: The spatial and functional diversity of cells in tissues is influenced by their 3D microenvironment, lineage history, and intercellular interactions. Technologies illustrated include: 1) 3D serial tissue sectioning combined with DNA nanoball arrays for high‐resolution spatial transcriptomics (e.g. Stereo‐seq) to resolve cellular positioning and gene expression at subcellular resolution. 2) seqFISH+ and Spateo for mapping gene expression and lineage trajectories during development and disease progression. 3) AI‐assisted spatial reconstruction to computationally integrate imaging and multi‐omics data for reconstructing cancer–immune cell interactions in the tumour microenvironment. Above tools reveal dynamic cell–cell communication, spatial heterogeneity, and the role of the microenvironment in shaping cell states and functions. Key technologies enabling SST‐cell characterisation, including single‐cell RNA sequencing (scRNA‐seq), Stereo‐seq, seqFISH+, Spateo, multi‐omics integration, and spatial imaging with artificial intelligence (AI)‐guided reconstruction. The SST‐cell framework highlights a paradigm shift in spatial cell biology, enabling detailed mechanistic insights into cell differentiation, organogenesis, cancer progression, and immune regulation. By integrating structural, functional, and molecular dimensions in space and time, SST‐cell analysis offers a transformative approach for understanding complex biological systems and advancing molecular medicine.

## 1 STEREOLOGICALLY SPATIOTEMPORAL CELL DEFINITION

To distinguish this from conventional one‐ or two‐dimensional spatialization, we define the concept of the “stereologically spatiotemporal cell” (SST‐cell) to describe the precise three‐dimensional localisation and interactions of intra‐ and extracellular components at the single cell level. We propose that understanding the SST‐cell represents a new frontline in clinical single‐cell biomedicine, providing new insights for the clinical translation and application of molecular medicine.^3^ Changes in intracellular components result in a high complexity of intercellular heterogeneity and multidimensional dynamics, posing challenges for real‐time monitoring and reproducibility. One of the major challenges is to accurately delineate and interpret the complex multidimensional structures and functions within and surrounding a single cell. Due to the difficulty in detecting the dynamic changes of intracellular components, it remains challenging to uncover and monitor the phenotypic diversity, autonomous responses, and metabolic functions of cellular components in their precise stereologically spatiotemporal contexts. A new technology for the genetic tracing codes of distinct endodermal regions was recently developed by integrating high‐throughput, high‐precision single‐cell RNA sequencing with sophisticated imaging.^4^ This approach uncovered the spatiotemporal and genetic lineage differentiation of endodermal cells at single‐cell resolution. The spatiotemporal trajectories and dynamic rearrangement of each single cell during early endodermal organogenesis were shown to be influenced by the three‐dimensional extracellular microenvironments and intercellular communication. These findings imply that stereological and spatiotemporal regulation may play an important role in organ development via cell recruitment or differentiation from multiple origins. The intra‐ and extra‐SST‐cell can be comprehensively presented using continuous spatial transcriptomic data from tissue sections, such as that obtained by Stereo‐seq, combined with computational mapping at single‐cell resolution.^5^ The current Perspective is focusing on the importance of intra‐ and extracellular stereoscopic changes in SST‐cells, the technological advances enabling their precise detection of intracellular positional changes, and their implications for the diagnosis and treatment of human diseases.

## 2 THREE‐DIMENSIONAL CHROMATIN

Spatiotemporal changes in three‐dimensional (3D) chromatin architecture are important for determining transcriptional regulations, genetic epigenetics, and the transmission of genetic information within SST‐cells. Technological advancements have deepened our understanding of the density and structural changes of 3D chromatin. The dynamic transformation of its spatial structure changes gene expression, which plays an important role in the development and regulation of genetic diseases. The 3D construction of chromosomes ensures their proper formation and spatial positioning, facilitates the recruitment of regulatory factors, and underlies the mechanisms by which these factors maintain chromatin architecture. This 3D chromatin architecture can be disrupted by the dysfunction of mediators of replication and DNA double‐strand breaks, and impair topologically associating domains rather than DNA synthesis itself, leading to unintended replication events in damaged chromatin and increased DNA damage, particularly in cancer cells.^6^ During the cell cycle, the spatiotemporal architecture of chromatin is dynamically regulated by interactions among structural maintenance of chromosome complexes,^7^ including chromatin loop‐extruding cohesin, sister chromatid‐cohesive cohesion, and mitotic chromosome‐associated condensins. This 3D chromatin organisation plays a crucial role not only for maintaining transcriptomic network integrity and nuclear shape but also for influencing the spatiotemporal locations of subcellular organelles and the overall structure of the SST‐cells. Recently, a new sequencing‐based method named Linking mRNA to Chromatin Architecture has enabled the simultaneous measurement of the single‐cell 3D genome architecture in the nucleus and transcriptomic profiles in the cytoplasm of the same cell.^8^ This powerful tool in single‐cell measurements precisely captures high‐order chromatin structure, comprehensively provides the transcriptomic profiles, accurately distinguishes cell types based on chromatin interactions and gene expression, dynamically examines the role of gene positioning in expression, and systematically defines continuous cell‐state trajectory during development. Spatiotemporal alterations in 3D chromatin architecture can cause multiple disorders, including defects in organ development, aberrant neural connectivity, carcinogenesis, and cancer progression.

## 3 SPATIAL ORGANELLE INTERACTOMES

A large number of intracellular organelles and sub‐organelles, along with their intercommunications, decide cellular biological types, subtype specification, and type‐specific functions. The cellular and molecular phenomes and functions of a cell are fully dependent upon the dynamics of organelle numbers, volumes, speeds, positions, and dynamic inter‐organelle contacts, particularly among membrane‐bound organelles. Most organelles and sub‐organelles are identified using specific protein markers that define their identity, specificity, spatial separation, and abundance in response to microenvironmental or pathological stimuli. The set of all organelle interactions, termed the organelle interactome, performs molecular exchange and signal transductions, either through direct membrane contacts or proximity‐based interactions among organelles. Using two‐layer DNA seqFISH+, a method that simultaneously detects genomic loci, transcriptomes, and subnuclear structures in the same single cell, Takei et al. demonstrated that repressive chromatin or heterochromatin regions as part of the subnuclear compartments vary in a cell‐type‐dependent pattern.^9^ Within the subnuclear interactome, both RNA polymerase II‐enriched and speckle‐associated regions showed cell‐type‐specific gene expression, where the former are locally associated with long, sparsely distributed genes, and the latter with short, densely packed genes. High‐resolution, single‐cell multi‐omic technologies that enable the simultaneous observation of subnuclear structures, corresponding genomic regions, and regulated gene expression in a single cell within complex tissues provide new insights into the mechanisms of gene dysregulations, misexpressions, and dysfunctions in diseases. The characteristic distribution and dispersion pattern of each organelle in three‐dimensional space and a reproducible pattern of contacts among organelle interactomes can be changed in response to pathogens, carcinogens, and therapies. Among these, various shapes and functions of endoplasmic reticulum (ER) are hardly monitored with dynamical morphology and the cytoplasmic flows in the cell. The adhesion site disassembly, actin dynamics, and ER‐plasma membrane polarisation may be regulated by the ER‐actin tether‐oriented calcium signalling factor and calcium signalling near ER‐actin interfaces.^10^ It is possible that those morphological sub‐domains/sub‐organelles of the ER are prepared for contacting and functioning with other organelles. These mitochondrial and lysosomal dynamics and intercommunications are carried out and regulated through active, GTP‐bound lysosomal RAB7, and can be disconnected by the RAB7 GTPase‐activating protein TBC1D15, or fissioned by lysosomal RAB7 hydrolysis via TBC1D15.^11^ Interventions of organelle interactomes can be a new alternative to the discovery of diagnostic biomarkers and therapeutic targets.

## 4 SST‐CELL MULTI‐OMICS

Multi‐dimensional dynamics of intra‐ and extracellular components decide the cell performances in the tissue and organ functions in the body. The development of technologies such as continuous Stereo‐seq, a combination of DNA nanoball‐patterned arrays and in situ RNA capture, allows the 3D locations and contacts of intra‐and extracellular components to be defined with high‐spatial resolution omics sequencing. This spatial omics‐sequencing approach enables the detailed dissection of spatial heterogeneity among different cell subtypes and states, as well as their intercellular communications.^5^ Using continuous spatial transcriptomics, their 3D lineage trajectories of intra‐ and extra‐embryonic molecular and cellular components have been traced, revealing how their interactions contribute to early development.^12^ The development of the human embryo critically depends upon characters and patterns of 3D spatial arrangements of those cells, while the intracellular transcriptomic regulations orient their positioning and function of these cells. By integrating experimental and computational tools for the efficient and specific capture of continuous spatial transcriptomes, the metastatic capacity of a tumour has been found to be highly dependent on the spatial rearrangements and contacts of various cell types within the tumour microenvironment.^13^ This spatially resolved approach provides a potential to determine the stereological spatialization of intra‐ and extracellular components, map 3D cell‐cell interactions with simultaneous states of transcriptomic regulatory networks, and explore the molecular mechanism based on cellular spatial re‐locations. A spatial atlas of the human thymus, for example, has demonstrated the full 3D trajectory of T cell lineage differentiation at the beginning of the second trimester of fetal development, including cytokine and chemokine expression patterns, the spatial shift of thymic epithelial cell populations and subtypes between medullary and cortical regions, and development of CD4 and CD8 thymocytes.^14^ In tumours, the stereological spatialization of tumour microenvironment provides 3D locations, contact interface, and communication networks among cancer cells, cancer‐associated cells, immune cells, and signalling mediators. These insights are obtained by measuring the correlations between average module expression, sample purity, and spatial metrics such as the relative distance between centroid and periphery.^15^ In contrast to 1D and 2D spatial transcriptomics, 3D spatial profiling of the tumour microenvironment exhibits more specific and precise interactions among activated cells, population trajectories, cell subtypes, and spatial proclivities of interactomes and signalling pathways. Moreover, the volume of the tumour microenvironment is spatially divided into connectivity, loop, and microregion of tumour growth patterns and interactomes. Spatial distributions of 3D tumour volume decide the nature of cancer cell identity and metastasis.^16^

## 5 CHALLENGES AND POTENTIALS

The SST‐cell is a new and important frontline of molecular medicine, an unexplored resource for uncovering diagnostic biomarkers and therapeutic targets, and an emerging discipline of biomedicine. A major challenge is to precisely define and dynamically monitor the stereological spatialization and its changes of intra‐ and extracellular components. Although organelle‐specific marker proteins can help evaluate the interactions between them, it remains technically difficult to accurately define the 3D locations and distances between organelles, especially at the same time point these interactions occur. There is an urgent need to develop sub‐organelle‐specific antibody panels for distinguishing between sub‐organellar compartments of the organelle and to map their multidirectional spatial orientations within the cell. In addition to the cost burden of continuous stereo‐seq, standardised analyses and graphical representations in 3D are critical to overcome the overlap of data points and patterns, due to the high‐throughput manner of spatial multi‐omics. New, precise, and repeatable methodologies should be established to address the multilayered spatial organisation and functions of intracellular and intraorganellar molecular components. Further studies on human cellular and organellar interactomes are expected, especially in pathological contexts. Resolving the 3D spatial locations and functional states of intracellular components at single‐cell resolution remains a particularly formidable challenge.

The clinical translation of SST‐cell measurements requires a clear cell identity annotation, simplified experimental workflows, artificial intelligence‐driven high‐throughput analysis, uniform sampling, adaptability to diverse cell sizes, and broad technical extensibility. To this end, a spatially enhanced resolution single‐cell sequencing platform, Stereo‐cell, is developed. Based on high‐density DNA nanoball‐patterned arrays, it is able to measure cell surface proteins, morphologies, and transcriptomic profiles simultaneously.^17^ In addition to using marker gene panels for annotation, this platform allows scattered cells seeded on poly‐L‐lysine‐coated array chips to be stained and labelled with biochemicals or target‐specific antibodies, similar to routine applications in clinical haematology. This system integrates imaging‐based measurements with molecular multi‐omic profiles and can accommodate extracellular vesicles, microstructures, large multinucleated cells, and other complex clinical specimens for in situ multi‐omic profiling.

A deep understanding of cellular and organellar interactomes and their real‐time communication requires monitoring the stereological spatializations of intracellular components in live cells. A system of CRISPR‐mediated transcriptome organisation (CRISPR‐TO)was developed for real‐time monitoring of the spatial dynamics of RNA location across various subcellular compartments in living cells.^18^ CRISPR‐TO couples the dCas13 unit (fused with one ABA dimerisation domain) with the signal unit (subcellular localisation signal or motor protein fused with the other dimerisation domain) through the gRNA unit (chemical‐inducible dimerisation), to perform the programmable control of endogenous RNA localisation in live cells. CRISPR‐TO is applicable in primary human cells, supports multiplex detection of RNA localisations to investigate cooperative roles, and is capable of programmability to screen high‐throughput functions. In addition to RNA levels, a “zero‐distance” photo‐crosslinking approach has been developed to identify the proteome that physically interacts with DNA in living cells.^19^ This creates new opportunities to define the manner of protein‐DNA interactions, maintenance of 3D chromatin architecture, and link the stereological spatialization of genomic regulation, and relations with cellular and molecular phenomes and functions.

In conclusion, cell function and morphology are highly dependent upon the stereologically spatiotemporal positioning, precise dimensions, and interactions of subcellular organelles. The stability of the 3D chromatin architecture, along with spatial intra‐ and extracellular and organellar interactomes, governs cell differentiation and maintains the bioecology of microenvironments. The multi‐omic profiles and locations in an SST‐cell can be defined by combining image‐ and molecular omics‐based strategies and by monitoring the real‐time dynamics of spatial transcriptomes and proteomics, although technical hurdles remain. SST‐cell biology provides a transformative new perspective for understanding pathogenesis and opens new alternatives to the discovery of biomarkers for diagnosis and drug development.

## References

[1] Hein MY , Peng D , Todorova V , et al. Global organelle profiling reveals subcellular localization and remodeling at proteome scale. Cell. 2025;188(4):1137‐1155. doi:10.1016/j.cell.2024.11.028. e20. 39742809

[2] Martinez‐Val A , Bekker‐Jensen DB , Steigerwald S , et al. Spatial‐proteomics reveals phospho‐signaling dynamics at subcellular resolution. Nat Commun. 2021;12:7113. doi:10.1038/s41467-021-27398-y 34876567 PMC8651693

[3] Wang X , Duan W , Liu X , Fan J . An important step to translate single‐cell measurement into clinical practice: stereoscopic cells. Clin Transl Med. 2025;15:e70304. doi:10.1002/ctm2.70304 40226973 PMC11995416

[4] Li KR , Yu PL , Zheng QQ , et al. Spatiotemporal and genetic cell lineage tracing of endodermal organogenesis at single‐cell resolution. Cell. 2025;188(3):24. doi:10.1016/j.cell.2024.12.012 39824184

[5] Chen A , Liao S , Cheng M , et al. Spatiotemporal transcriptomic atlas of mouse organogenesis using DNA nanoball‐patterned arrays. Cell. 2022;185(10):1777‐1792.e21. doi:10.1016/j.cell.2022.04.003 35512705

[6] Zhao H , Shu L , Qin S , et al. Extensive mutual influences of SMC complexes shape 3D genome folding. Nature. 2025;640(8058):543‐553. doi:10.1038/s41586-025-08638-3 40011778 PMC12726995

[7] Sebastian R , Sun EG , Fedkenheuer M , et al. Mechanism for local attenuation of DNA replication at double‐strand breaks. Nature. 2025;639(8056):1084‐1092. doi:10.1038/s41586-024-08557-9 39972127

[8] Wu H , Zhang J , Jian F , et al. Simultaneous single‐cell three‐dimensional genome and gene expression profiling uncovers dynamic enhancer connectivity underlying olfactory receptor choice. Nat Methods. 2024;21(6):974‐982. doi:10.1038/s41592-024-02239-0 38622459 PMC11166570

[9] Takei Y , Yang Y , White J , et al. Spatial multi‐omics reveals cell‐type‐specific nuclear compartments. Nature. 2025;641(8064):1037‐1047. doi:10.1038/s41586-025-08838-x 40205045

[10] Merta H , Gov K , Isogai T , et al. Spatial proteomics of ER tubules reveals CLMN, an ER‐actin tether at focal adhesions that promotes cell migration. Cell Rep. 2025;44(4):115502. doi:10.1016/j.celrep.2025.115502 40184252 PMC12416146

[11] Wong YC , Ysselstein D , Krainc D . Mitochondria‐lysosome contacts regulate mitochondrial fission via RAB7 GTP hydrolysis. Nature. 2018;554(7692):382‐386. doi:10.1038/nature25486 29364868 PMC6209448

[12] Xiao Z , Cui L , Yuan Y , He N , Xie X , Lin S , et al. 3D reconstruction of a gastrulating human embryo. Cell. 2024;187(11):2855‐2874.e19. doi:10.1016/j.cell.2024.03.041 38657603

[13] Schott M , León‐Periñán D , Splendiani E , et al. Open‐ST: high‐resolution spatial transcriptomics in 3D. Cell. 2024;187(15):3953‐3972.e26. doi:10.1016/j.cell.2024.05.055 38917789

[14] Yayon N , Kedlian VR , Boehme L , et al. A spatial human thymus cell atlas mapped to a continuous tissue axis. Nature. 2024;635(8039):708‐718. doi:10.1038/s41586-024-07944-6 39567784 PMC11578893

[15] Mathur R , Wang Q , Schupp PG , et al. Glioblastoma evolution and heterogeneity from a 3D whole‐tumor perspective. Cell. 2024;187(2):446‐463.e16. doi:10.1016/j.cell.2023.12.013 38242087 PMC10832360

[16] Mo CK , Liu J , Chen S , et al. Tumour evolution and microenvironment interactions in 2D and 3D space. Nature. 2024;634(8036):1178‐1186. doi:10.1038/s41586-024-08087-4 39478210 PMC11525187

[17] Liao S , Zhou X , Liu C , et al. Stereo‐cell: spatial enhanced‐resolution single‐cell sequencing with high‐density DNA nanoball‐patterned arrays. Science. 2025;389(6762):eadr0475. doi:10.1126/science.adr0475 40839715

[18] Han M , Fu ML , Zhu Y , et al. Programmable control of spatial transcriptome in live cells and neurons. Nature. 2025;643(8070):241‐251. doi:10.1038/s41586-025-09020-z 40399675 PMC12882822

[19] Trendel J , Trendel S , Sha S , et al. The human proteome with direct physical access to DNA. Cell. 2025;188(16):S0092‐8674(25)00507‐0. doi:10.1016/j.cell.2025.04.037 40409270

